# Correlation between the Long Head of the Biceps Microscopic Degeneration and Extent of Apoptotic Process

**DOI:** 10.3390/jcm13154520

**Published:** 2024-08-02

**Authors:** Łukasz Jaworski, Jan Zabrzyński, Peter J. Millett, Marco-Christopher Rupp, Filippo Familiari, Gazi Huri, Jakub Erdmann, Michał Błachowski, Przemysław Pękala, Maciej Gagat

**Affiliations:** 1 Department of Orthopaedics and Traumatology, Faculty of Medicine, Collegium Medicum in Bydgoszcz, Nicolaus Copernicus University in Toruń, 85-092 Bydgoszcz, Poland; lukaszmjaworski@gmail.com (Ł.J.); zabrzynski@gmail.com (J.Z.); blachowskiort@gmail.com (M.B.); 2 Steadman Philippon Research Institute, The Steadman Clinic, Vail, CO 81657, USA; drmillet@thesteadmanclinic.com; 3 Department of Sports Orthopaedics, Technical University of Munich, 80333 Munich, Germany; marco.rupp@tum.de; 4 Department of Orthopaedic and Trauma Surgery, “Mater Domini” University Hospital, “Magna Græcia” University, 88100 Catanzaro, Italy; filippofamiliari@unicz.it; 5 Department of Orthopaedics and Traumatology, Hacettepe Universitesi, Ankara 06800, Turkey; gazihuri@yahoo.com; 6 International Evidence-Based Anatomy Working Group, Department of Anatomy, Jagiellonian University Medical College, 31-008 Krakow, Poland; pekalapa@gmail.com; 7 Department of Histology and Embryology, Faculty of Medicine, Nicolaus Copernicus University in Torun, 87-100 Torun, Poland; mgagat@cm.umk.pl; 8 Collegium Medicum, Mazovian Academy in Płock, 09-402 Płock, Poland

**Keywords:** LHBT, tendon, tendinopathy, Bonar score, apoptotic process, caspase-3, protein p53, BCL-2

## Abstract

**Objectives:** The purpose of this study was to determine the correlation between microscopic degeneration in the long head of the biceps tendon (LHBT) and the apoptotic process. **Methods:** This study included 26 consecutive patients who had undergone arthroscopic biceps tenodesis or tenotomy for symptomatic LHBT with or without concomitant rotator cuff tears (RCTs). Histological examination of the specimens under a light microscope was conducted after staining with hematoxylin, eosin, and the Alcian blue. Histopathological changes were assessed using the original Bonar score and the modified Bonar score and then correlated with the expression of the subsequent apoptosis markers: activated caspase-3 (casp3), tumor protein p53 (p53), and B-cell lymphoma 2 (BCL-2). **Results:** The mean original Bonar score was 8.65 (range 5–11), while the modified Bonar score was 7.61. There was no correlation between the original Bonar score and the age of the patients, but a positive correlation was found between the modified Bonar score and the age of the patients (*p* = 0.0022). There was no correlation between the age of patients and the expression indexes of BCL-2 and casp3. However, the expression of the p53 index showed a positive correlation with patient aging (*p* = 0.0441). Furthermore, there was no correlation observed between the expression of apoptotic indexes and both the original and modified Bonar scale. **Conclusions:** In LHB tendinopathy, the expression of apoptosis does not seem to directly correlate with the extent of degeneration, particularly in the late stages of tendinopathy. However, the transformations observed in collagen and ground substance were significantly associated with age, as well as tendinous tissue degeneration quantified according to modified Bonar score. The age of patients was also linked with the expression of the p53 index, as an increased apoptosis in the studied population.

## 1. Introduction

Tendinopathy is a chronic disorder affecting tendons, characterized by an immune response dysregulation with an early-phase inflammatory response phenotype which clinically manifests in impaired tendon healing and excessive degeneration [1,2,3]. Despite recent advancements in understanding tendinous tissue, the exact causes of tendinopathy are still a subject of debate. Microscopically, tendinopathy is marked by disruptions in collagen architecture, the accumulation of proteoglycans, changes in tenocyte morphology and population, and the expansion of neovessels [4,5,6,7]. In the setting of tendon metabolism, apoptosis has been observed both in the natural healing process of tendinous tissue and in pathological tendinopathy [8,9]. Following a tendon lesion, the healing process is marked by a brief inflammatory phase that typically lasts for 7–10 days. Increased apoptosis during tendon healing may serve as a mechanism to restore tissue homeostasis after the repair process [9]. Conversely, Yuan et al. proposed that excessive apoptosis is the primary cause of tendinopathy which leads to tendon lesions as a secondary manifestation of degeneration [10], reflecting the homogeneity in the literature on the role of apoptosis in the setting of tendinopathy. Specific to tendinopathy, the process of apoptosis can be triggered by multiple external and internal stimuli [11].

LHB tendinopathy typically arises due to repetitive and chronic traction and friction forces that impact the tendinous tissue, resulting in microtrauma during movement of the shoulder joint. In particular, the intra-articular portion, which runs beneath the rotator cuff and attaches to the superior labrum and glenoid, is prone to injury, especially in conjunction with the degeneration of adjacent structures like the rotator cuff tendons and biceps reflection pulley. Such co-existing damage can exacerbate the pathological processes at play [12]. Despite these observations, the apoptotic profile in the setting LHBT and its role remains enigmatic and has not been thoroughly explored in comparison to the rotator cuff tendons [11,13,14].

The aim of this study was to investigate the correlation between the degree of microscopic degeneration in the long head of the biceps tendon during chronic tendinopathy and the apoptotic process. Our hypothesis proposed that the degeneration of the tendinous tissue, as evaluated using both the original Bonar score and the modified Bonar score, would correspond to the extent of the apoptotic process [15].

## 2. Materials and Methods

### 2.1. Ethics and General Characteristics

The study was conducted in accordance with the principles outlined in the Declaration of Helsinki for experiments involving human subjects. Prior to the commencement of the study, approval was obtained from the Nicolaus Copernicus Bioethics Committee (approval number KB 598/2016; the approved date 20 September 2016). A total of 26 patients who had undergone shoulder arthroscopy were included in the study cohort due to symptomatic LHB tendinopathy and associated shoulder lesions between 2016 and 2017. Long head of the biceps surgery was typically performed as part of arthroscopic surgery. All participants included in the study were preoperative diagnosed with LHBT tendinopathy, which was based on a clinical examination including tests such as tenderness over the bicipital groove, Speed test, Yergason test, Abbot-Saunders test, as well as non-contrast magnetic resonance scans (MRI) of the shoulder. The inclusion criteria required a lack of response to conservative treatment, which included a minimum of three months of physiotherapy, and a minimum age of 18 years. Exclusion criteria encompassed a history of systemic inflammatory or rheumatic diseases, previous shoulder surgical treatment, or corticosteroid injections within the year prior to the surgery. Written informed consent was obtained from all patients prior to their participation in the study.

### 2.2. Operative Treatment

All patients underwent shoulder arthroscopy using a standard 30° arthroscope from Smith & Nephew while positioned in the beach chair position, under general anesthesia or brachial plexus anesthesia. The intracapsular portion of the LHBT was evaluated by probe from its insertion to the bicipital groove to assess for tears and swelling. The condition of the biceps pulley, stability of the LHBT in the groove, and the extracapsular part of the tendon were also examined. The arthroscopic examination and diagnosis of LHBT tears were made based on the Lafosse classification, which includes grade 0 (normal tendon), grade I (minor lesion), and grade II (major lesion). Subsequently, all patients underwent either arthroscopic tenodesis or tenotomy procedures, depending on the surgeon’s decision, followed by the excision of the intracapsular portion of the LHBT to obtain a sample for further histological study. All patients underwent either biceps tenodesis or tenotomy, depending on the surgeon’s decision, which was based on clinical data; specifically, those for tenodesis were qualified patients who did not accept a possible Popeye deformity, and those for tenotomy were patients who were older than 50 years and who accepted a possible arm deformity. Any concurrent lesions were addressed through arthroscopic techniques. Concomitant rotator cuff tears were classified according to the Snyder classification. The letter indicates the location of the rotator cuff tear (A—articular side; B—bursal side; C—full-thickness tear connecting A and B), while the number indicates the severity of tear. The following were included in partial tears (A and B): grade 0 (normal cuff), grade 1 (minimal, superficial), grade 2 (moderate, fraying fibers < 2 cm), grade 3 (more severe, fraying and fragmentation of the fibers < 3 cm), and grade 4 (very severe, fraying and fragmentation of the fibers with a sizable flap tear). The following were included in complete tears (C): grade 1 (small, but complete tear), grade 2 (moderate tear < 2 cm), grade 3 (large tear with minimal retraction, 3–4 cm), and grade 4 (massive tear, associated with retraction and scarring of remaining tendon).

### 2.3. Microscopic Examination of the LHBT Specimens

The resected tendon fragment was preserved in a fresh and sterile 10% buffered formalin solution. After 24 h, it was dehydrated and embedded in paraffin, enabling further histological evaluation. The samples were prepared using the hematoxylin and eosin (H&E) staining method, as well as the Alcian blue protocol, and were examined under light microscopy using 5 μm sections. Alcian blue staining was employed to specifically assess the presence of ground substance glycosaminoglycans. The microscopic evaluation was carried out by two experienced observers who specialize in tendinous tissue and were blinded to the identity of the samples. The extent of histopathological changes was assessed based on the original assumptions of the original Bonar score [16]. This scoring system evaluates four main variables: tenocyte morphology, accumulation of ground substance elements, neovascularity, and collagen architecture. An ascending scoring range of 0 to 3 points was assigned to each variable, with 0 indicating normal tissue and 3 representing extreme pathology. A completely normal tendon would have a score of 0, while a severely degenerated tendon would score 12. Additionally, in the second step of the examination, LHBT specimens were evaluated using the modified Bonar score developed by Zabrzyński et al. [15]. In this modified scoring system, the attributes of the neovascularization variable in the original Bonar scale were reversed. A score of three points was assigned to a normal tendon with a minimal occurrence of blood vessels (absent neovascularization), two points for the incidental presence of capillary clusters of less than one per 10 high-power fields (HPFs; mild neovascularization), one point for 1–2 clusters per 10 HPFs (moderate neovascularization), and zero points for more than two clusters per 10 HPFs (abundant neovascularization). This modified scoring system acknowledges the complex role of neovascularization in tendinopathy [15].

For immunohistochemical staining, the sections were deparaffinized, rehydrated, and the activity of endogenous peroxidase was blocked using 3% hydrogen peroxide. Staining was performed using the BenchMark Ultra system from Ventana Medical Systems (Roche) from Risch-Rotkreuz, Switzerland, following the manufacturer’s instructions. The apoptotic response in tendon samples was assessed by evaluating the density of apoptotic cells, specifically examining antibodies such as activated caspase-3, protein p53, and BCL-2. The evaluation of apoptotic response and immunohistochemical analysis was conducted by two experienced observers specialized in tendinous tissue, who were blinded to the identity of the samples. The density of apoptotic cells was classified based on the number of positive cells observed in the specimen, with 0 points indicating no positive cells, 1 point denoting less than 10 positive cells per specimen, and 2 points representing more than 10 positive cells per specimen.

### 2.4. Correlation Analyses

The results of the original as well as modified Bonar scores were correlated to patient age as well as the expression of the apoptosis markers activated caspase-3, protein p53, and BCL-2.

### 2.5. Statistical Analysis

All group comparisons and statistical analyses were conducted by two independent investigators using GraphPad Prism software (GraphPad 8.0.1 Software, Dotmatics, UK). A *p*-value less than 0.05 was considered statistically significant. The normality of variables was assessed using the Shapiro–Wilk test. Relationships between the studied parameters were evaluated using the Spearman’s rank correlation coefficient. According to the non-normal distribution of the data, intergroup comparisons were performed using non-parametric tests, specifically the Mann–Whitney U test for comparing two groups.

## 3. Results

A total of 26 patients who had undergone shoulder arthroscopy were included in the study cohort due to chronic LHB tendinopathy and associated shoulder lesions. Among the patients examined, arthroscopic LHB tenodesis was performed on 19 patients, while tenotomy was performed on 7 patients. Furthermore, all of the patients underwent other arthroscopic procedures, including rotator cuff tear repairs (Snyder classification C-2) in seven patients and repairs for massive rotator cuff tears (Snyder classification C-4) in nine patients. Ten patients were diagnosed with a subacromial impingement with rotator cuff tendinopathy (Snyder classification B-1 and B-2 changes in rotator cuff), and they underwent arthroscopic debridement with subacromial decompression. All of the LHB tendon lesions were classified as grade II according to the Lafosse classification. The average follow-up period after surgery was 40.9 months, ranging from 24 to 65 months, and no reoperations were reported during this period. The mean age of the patients included in the study cohort was 51.5 years, ranging from 28 to 74 years. The summary of population characteristics and results is shown in Table 1.

### 3.1. Histological Examination

**Figure 1:**
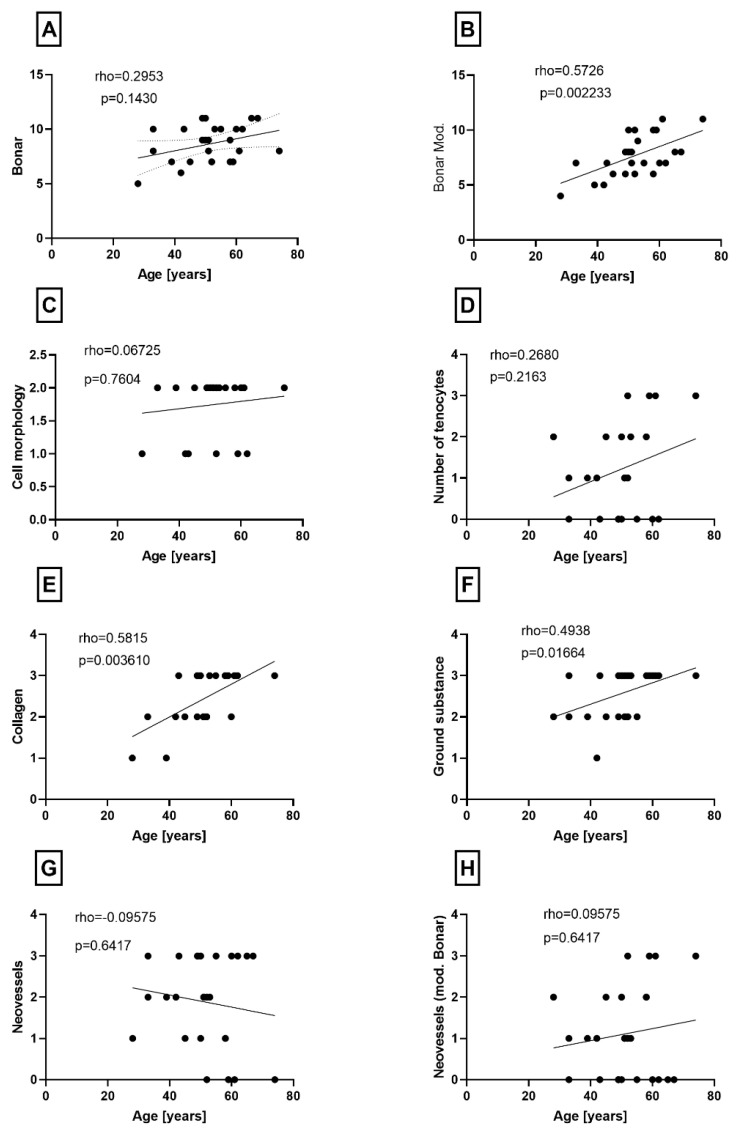
(**A**) Correlation between Bonar and (**B**) modified Bonar scores and age. Correlation between (**C**) tenocyte morphology, (**D**) number of tenocytes, (**E**) transformations observed in collagen, (**F**) transformations observed in ground substances and age. Correlation between (**G**) expansion of neovessels, (**H**) and neovessels according to modified Bonar scale and age.

Histological examination of the specimens under a light microscope revealed degeneration of the tendinous tissue in all cases. The mean original Bonar score was 8.65 (range 5–11), while the modified Bonar score developed by Zabrzyński et al. [15] was 7.61 (range 4–11). There was no correlation between the original Bonar score and the age of the patients (Figure 1A). However, a positive correlation was found between the modified Bonar score and the age of the patients (*p* = 0.0022) (Figure 1B). Furthermore, when analyzing the individual components of the original and modified Bonar scales, a statistically significant correlation with age was observed in terms of collagen and ground substance accumulation variables (*p* = 0.0036 and *p* = 0.0166, respectively) (Figure 1E,F).

### 3.2. Immunohistochemical Analysis

Immunohistochemical analysis revealed positive reactions for BCL-2 in 12 patients, cleaved caspase 3 in 16 patients, and p53 in 12 patients. Among the BCL-2 reactions, moderate reactions (1 point) were observed in 8 out of 12 cases. For cleaved caspase 3 reactions, moderate reactions were seen in 5 out of 16 cases, while for p53 reactions, moderate reactions were observed in 10 out of 12 cases. Abundant reactions (2 points) were observed in 4 cases for BCL2, 11 cases for cleaved caspase 3, and 2 cases for p53.

**Figure 2:**
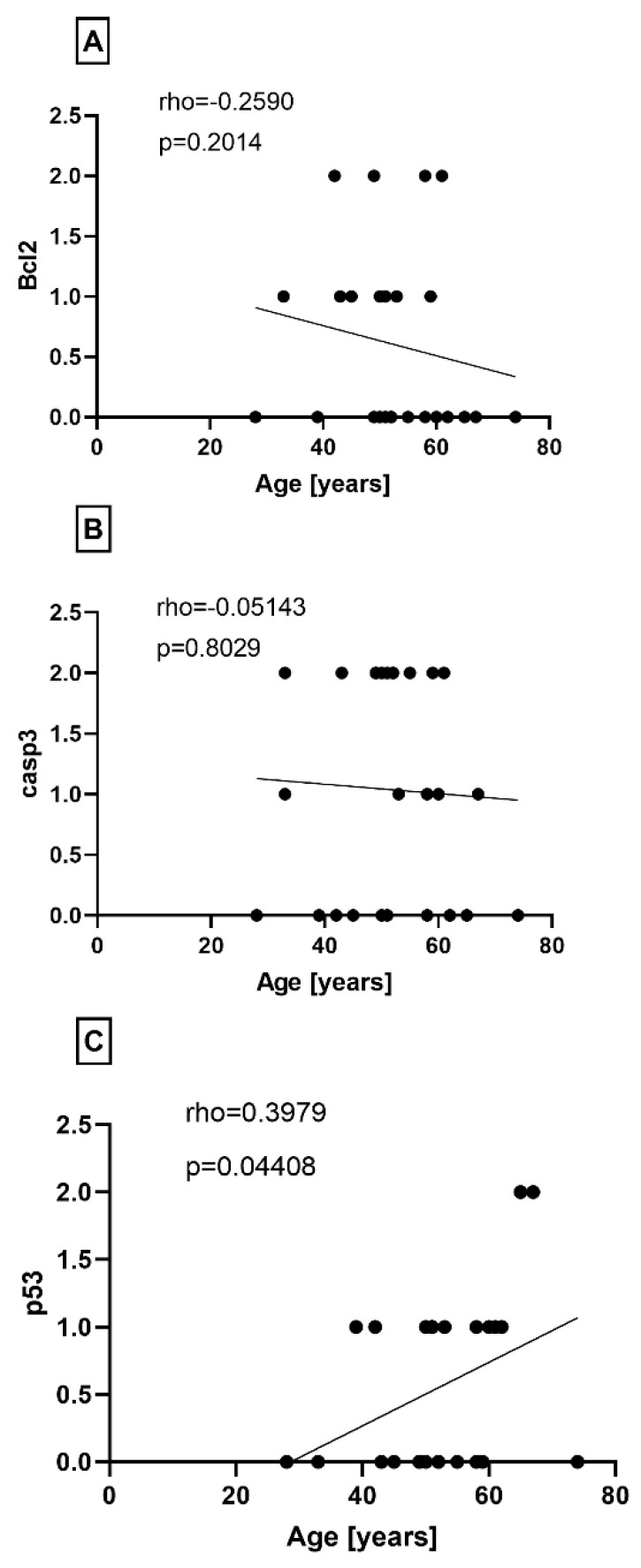
Correlation between the (**A**) BCL2, (**B**) cleaved caspase 3 index, and (**C**) p53 index and the age of patients.

**Figure 3:**
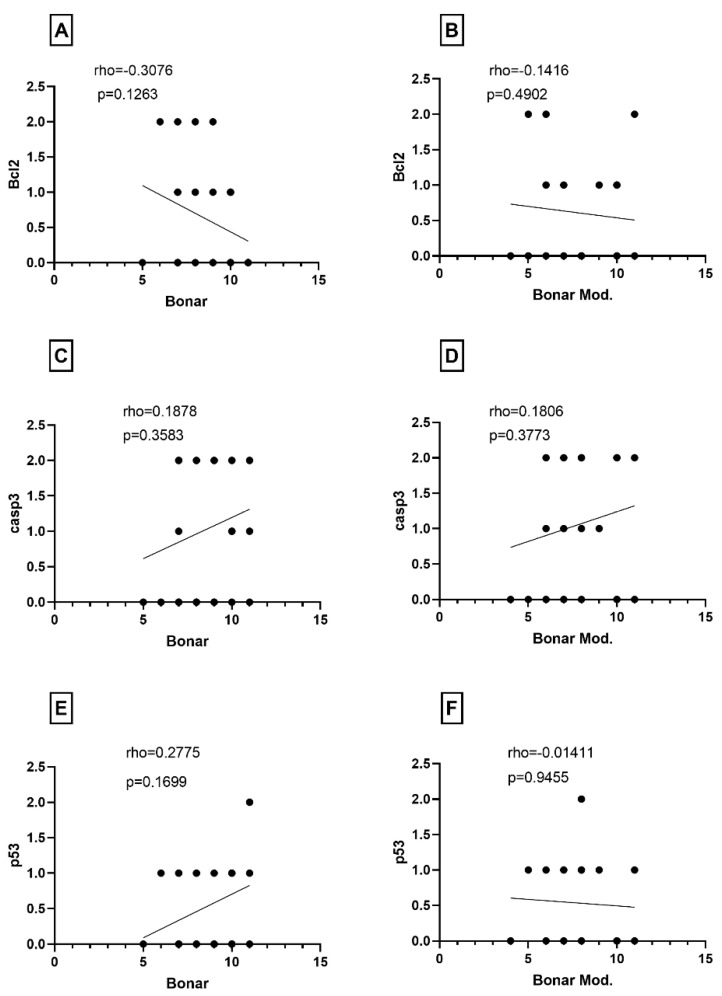
Correlation between the (**A**) BCL2 index, (**C**) cleaved caspase 3 index, and (**E**) p53 index and Bonar scale. Correlation between the (**B**) BCL2, (**D**) cleaved caspase 3 index, (**F**) p53 index and modified Bonar scale.

Further analysis of the relationship between age and apoptotic marker expression revealed no correlation between the age of patients and the expression indexes of BCL-2 and casp3 (Figure 2A,B). However, the expression of the p53 index showed a positive correlation with patient aging (*p* = 0.0441) (Figure 2C). Furthermore, there was no correlation observed between the expression of apoptotic indexes (BCL-2, cleaved caspase 3, p53) and both the original and modified Bonar scores (Figure 3). While the separated components of the Bonar scale (tenocyte morphology, collagen architecture, ground substance accumulation, and neovascularization) were analyzed for correlation with the studied apoptotic indexes, no statistically significant dependencies were found in the entire comparison.

## 4. Discussion

The most important finding of this study was that the primary hypothesis of this study was refuted. The level of tendinous tissue degeneration assessed by the commonly used Bonar score and its modification developed by Zabrzyński et al. [15] did not correlate with the expression of apoptotic markers such as casp3, p53, and BCL-2. However, the data indicate that modified Bonar score seemed to correlate with patient age better than the original version of this score. What is more, the rate of the apoptotic index of p53 was positively linked to age.

Although apoptosis is a natural physiological process that maintains tissue homeostasis, excessive apoptosis is often observed and associated with orthopedic degenerative disorders such as osteoarthritis, osteonecrosis of the femoral head, hip dysplasia and tendinopathies [8,17,18]. Numerous studies have reported that increased apoptotic cell death may be a feature of many tendinopathies such as LHB tendinopathy, rotator cuff tears, lateral epicondylitis, patellar tendinosis, and Achilles tendinopathy [6,8,10,19,20,21,22]. Hypoxia, excessive load, inflammation, and genetic predisposition are the main causes and risk factors for apoptosis in tendinous tissue [8,18]. The role of tenocytes in maintaining the balance of the extracellular matrix (ECM) and the bilateral dependencies involved are well established. Excessive and uncontrolled programmed cell death of tenocytes can disrupt the structure of the ECM, resulting in the loss of mechanical resistance in tendinous tissue. The amount of cells that undergo apoptosis or autophagic cell death increases with elevated levels of ECM disruption, which may indicate the creation of a self-amplifying cycle [6].

It is widely recognized that LHBT is a multifactorial process and there are different etiological theories, such as mechanical, vascular, apoptosis, and other [23]. Since programmed cell death seems to be a constant element of tendinopathy [6,8,10,19,20,21], it is logical to assume that the more advanced the tendinopathy is, the more apoptosis markers should be observed. Although, in this study, the relationship between the expression of apoptosis markers and the level of tendon degeneration does not appear to follow a positive correlation in the setting of LHB tendinopathy. This finding contradicts the conclusions drawn by Wu et al. [21]. They examined a group of patients of similar age and demonstrated that increased apoptotic index was positively correlated with a worsening histologic grade [21]. However, the method of measuring the intensity of apoptosis differed between their and our study. In their research, apoptotic cells were identified by labeling nuclear DNA fragments, while in this study, apoptosis markers were analyzed. There are no specific recommendations in the assessment of apoptosis and it may be advised to apply both methods in future studies [24]. Furthermore, in a study of Wu et al. [21], histological grading was based on a semiquantitative method that differs from the Bonar score. Based on our knowledge, no further studies have investigated the relationship between the level of induction of apoptosis and severity of tendinopathy. Available studies mainly focus on whether excessive apoptosis occurs in tendinopathy and do not investigate whether apoptosis intensity is associated with a worse histological degeneration [6,8,10,19,20].

In the present study, patients who had undergone prior corticosteroid injections were excluded. Interestingly, Puzzitello et al., in their systematic review, highlighted the potentially detrimental role of corticosteroid usage in increasing the number of apoptotic cells and decreasing cellular viability and metabolism [25]. They found that cellular viability was reduced 14 days after exposure to steroids, but not at 21 days after a single dose. Additionally, if a second application was administered within 7 or 14 days of the first, cellular viability did not return to normal levels. Moreover, exposure to steroids resulted in the loss of normal tenocyte and collagen morphology, as well as decreased vascular proliferation. From a biochemical standpoint, it was presented that glucocorticoid treatment may induce tenocytes’ senescence by inducing p53 expression and inhibition of sirtuin-1 [26]. What is more, glucocorticoids may stimulate the damage and death of rotator cuff tendon cells by inducing glutamate receptor NMDAR1 [27]. It is important to note that the abovementioned previous studies did not take into account whether glucocorticoids were injected in examined subjects, which could have introduced bias in the morphology and apoptosis rate of the tendinous tissue.

It is worth mentioning that p53 expression was observed in LHBT and was positively correlated with age. Activation of p53 due to various cellular stress (i.e., oxidative stress, DNA damage, and oncogenes activation) can inhibit cell proliferation by inducing senescence, apoptosis, or cell cycle arrest [28]. As age increases, oxidative stress accumulates in soft tissues which stimulates p53 activity and induces apoptosis-promoting tendon pathology. This simplified biochemical mechanism may be a hint explaining why many studies have proven that increased age affects physiological healing of the ruptured tendon and is correlated with tendon retears [29]. In this study, the activity of casp3 and BCL-2 did not correlate with age. Caspases’ activity remains a subject of debate, varies during aging, and differs between the tissues that are examined [30].

The Bonar score is commonly used to assess histopathological alterations in tendinous tissue across various tendons. The specific area of the tendon specimen to be investigated is not clearly defined, leading to variation in assessment methods among different authors. Some scientists assess the entire tendon sample, while others focus only on the most degenerated region [16,31]. In our study, we investigated the entire area of the tendon sample.

Several authors have attempted to modify the Bonar system by incorporating additional variables such as the number of inflammatory cells, cellularity/number of tenocytes, presence of calcifications, and cytoplasmic alterations in tenocytes [16,31,32]. The extent or level of the apoptotic process has not been previously incorporated into the Bonar system, which indicates a potential for expansion of this score, given the central role of apoptosis in the tendinopathy process. The apoptotic rate appears to be an independent variable that could enhance the existing morphological scoring systems. However, this study has not found an association between apoptosis markers and the severity of tendon pathology that may indicate no necessity of adding this parameter in the Bonar score. Another attempt to modify the Bonar scale focused on vascularization. For instance, a complete lack of vascularity is graded with zero points in the classical Bonar score, while in the modified Bonar score proposed by Zabrzyński et al., this microscopic finding is scored with 3 points [15]. They introduced the NDS (Neovascularization Degenerative Score), which was calculated based on the original Bonar system. In this modified scoring system, avascular tissue in pathological samples was assigned the highest grade of NDS (three points), rather than the lowest (zero points) [15]. Tendon degeneration primarily occurs in areas with poor blood supply, and hypoxia serves as a significant regulator of apoptosis. Regardless of the type of Bonar score used, we did not observe a clear link between vascularity and apoptotic rates in the examined samples. It is worth mentioning that in the current study, the correlation between age and Bonar score was not found, which is consistent with the results obtained by other authors [33,34]. Longo et al. presented histological evidence of minimal degenerative changes in LHBT at an advanced age who did not suffer from any LHB tendinopathy [35]. On the contrary, modified Bonar score was positively correlated with the age of the patients. There are studies that indicate reduced angiogenic potential associated with aging that may impair tendon healing and lead to more severe tendinopathy according to modified Bonar score [36].

This study has several limitations. First, the sample size was small, but comparable to previous similar studies [13,37,38]. Second, the age differences among individuals in our study may have influenced the expression of apoptotic indexes. Third, the presence of concomitant rotator cuff injuries of different severities and etiologies could introduce bias into the results. Fourth, a control group could not be included due to ethical considerations in our country.

## 5. Conclusions

In LHB tendinopathy, the expression of apoptosis does not seem to directly correlate with the extent of degeneration, particularly in the late stages of tendinopathy. However, the transformations observed in collagen and ground substance were significantly associated with age, as well as tendinous tissue degeneration quantified according to the modified Bonar score. The age of patients was also linked with the expression of the p53 index, as increased apoptosis in the studied population.

## Figures and Tables

**Table 1:** Table presenting population characteristics and histological results.

Population	Mean Age (Years)	Mean Follow-Up Period (Months)	Mean Bonar Score	Mean Modified Bonar Score	LHBT Arthroscopic Procedure	Macroscopic LHBT Classification According to Lafosse	Macroscopic RC Classification According to Snyder
*n* = 26	51. 8 (range from 28 to 74)	40.9 (range from 24 to 65)	8.65 (range 5–11)	7.61 (range 4–11)	LHB tenodesis in 19 patients LHB tenotomy in 7 patients	Grade II—26 patients	C-2 in 7 patients C-4 in 9 patients B-1 and B-2 in 10 patients

LHBT—long head of the biceps tendon; LHB—long head of the biceps; RC—rotator cuff.

## Data Availability

Data are unavailable due to privacy and ethical restrictions.

## References

[1] Abate M., Gravare Silbernagel K., Siljeholm C., Di Iorio A., De Amicis D., Salini V., Werner S., Paganelli R. (2009). Pathogenesis of Tendinopathies: Inflammation or Degeneration?. Arthritis Res. Ther..

[2] Fu S.-C., Rolf C., Cheuk Y.-C., Lui P.P., Chan K.-M. (2010). Deciphering the Pathogenesis of Tendinopathy: A Three-Stages Process. BMC Sports Sci. Med. Rehabil..

[3] Jomaa G., Kwan C.-K., Fu S.-C., Ling S.K.-K., Chan K.-M., Yung P.S.-H., Rolf C. (2020). A Systematic Review of Inflammatory Cells and Markers in Human Tendinopathy. BMC Musculoskelet. Disord..

[4] Andarawis-Puri N., Flatow E.L., Soslowsky L.J. (2015). Tendon Basic Science: Development, Repair, Regeneration, and Healing. J. Orthop. Res..

[5] Järvinen T.A. (2020). Neovascularisation in Tendinopathy: From Eradication to Stabilisation?. Br. J. Sports Med..

[6] Wu B., Chen J., Rosa T.D., Yu Q., Wang A., Xu J., Zheng M.-H. (2011). Cellular Response and Extracellular Matrix Breakdown in Rotator Cuff Tendon Rupture. Arch. Orthop. Trauma Surg..

[7] Zabrzynski J., Gagat M., Paczesny L., Grzanka D., Huri G. (2020). Correlation between Smoking and Neovascularization in Biceps Tendinopathy: A Functional Preoperative and Immunohistochemical Study. Ther. Adv. Chronic Dis..

[8] Benson R.T., McDonnell S.M., Knowles H.J., Rees J.L., Carr A.J., Hulley P.A. (2010). Tendinopathy and Tears of the Rotator Cuff Are Associated with Hypoxia and Apoptosis. J. Bone Jt. Surg. Br. Vol..

[9] Wu Y.F., Chen C.H., Cao Y., Avanessian B., Wang X.T., Tang J.B. (2010). Molecular Events of Cellular Apoptosis and Proliferation in the Early Tendon Healing Period. J. Hand Surg..

[10] Yuan J., Murrell G.A.C., Wei A.-Q., Wang M.-X. (2002). Apoptosis in Rotator Cuff Tendonopathy. J. Orthop. Res..

[11] Osti L., Buda M., Del Buono A., Osti R., Massari L., Maffulli N. (2017). Apoptosis and Rotator Cuff Tears: Scientific Evidence from Basic Science to Clinical Findings. Br. Med. Bull..

[12] Ditsios K., Agathangelidis F., Boutsiadis A., Karataglis D., Papadopoulos P. (2012). Long Head of the Biceps Pathology Combined with Rotator Cuff Tears. Adv. Orthop..

[13] Lundgreen K., Lian Ø., Scott A., Engebretsen L. (2013). Increased Levels of Apoptosis and P53 in Partial-Thickness Supraspinatus Tendon Tears. Knee Surg. Sports Traumatol. Arthrosc..

[14] Lundgreen K., Lian Ø.B., Scott A., Fearon A., Engebretsen L. (2014). 58 Smokers Have Worse Rotator Cuff Teartendon Degeneration And Apoptosis. Br. J. Sports Med..

[15] Zabrzyński J., Gagat M., Łapaj Ł., Paczesny Ł., Yataganbaba A., Szwedowski D., Huri G. (2021). Relationship between Long Head of the Biceps Tendon Histopathology and Long-Term Functional Results in Smokers. A Time to Reevaluate the Bonar Score?. Ther. Adv. Chronic Dis..

[16] Maffulli N., Longo U.G., Franceschi F., Rabitti C., Denaro V. (2008). Movin and Bonar Scores Assess the Same Characteristics of Tendon Histology. Clin. Orthop. Relat. Res..

[17] Hwang H.S., Kim H.A. (2015). Chondrocyte Apoptosis in the Pathogenesis of Osteoarthritis. Int. J. Mol. Sci..

[18] Wu P.-T., Su W.-R., Li C.-L., Hsieh J.-L., Ma C.-H., Wu C.-L., Kuo L.-C., Jou I.-M., Chen S.-Y. (2019). Inhibition of CD44 Induces Apoptosis, Inflammation, and Matrix Metalloproteinase Expression in Tendinopathy. J. Biol. Chem..

[19] Lian Ø., Scott A., Engebretsen L., Bahr R., Duronio V., Khan K. (2007). Excessive Apoptosis in Patellar Tendinopathy in Athletes. Am. J. Sports Med..

[20] Pearce C.J., Ismail M., Calder J.D. (2009). Is Apoptosis the Cause of Noninsertional Achilles Tendinopathy?. Am. J. Sports Med..

[21] Wu P.-T., Jou I.-M., Yang C.-C., Lin C.-J., Yang C.-Y., Su F.-C., Su W.-R. (2014). The Severity of the Long Head Biceps Tendinopathy in Patients with Chronic Rotator Cuff Tears: Macroscopic versus Microscopic Results. J. Shoulder Elb. Surg..

[22] Chen L., Zheng J.J.Y., Li G., Yuan J., Ebert J.R., Li H., Papadimitriou J., Wang Q., Wood D., Jones C.W. (2020). Pathogenesis and Clinical Management of Obesity-Related Knee Osteoarthritis: Impact of Mechanical Loading. J. Orthop. Transl..

[23] Raney E.B., Thankam F.G., Dilisio M.F., Agrawal D.K. (2017). Pain and the Pathogenesis of Biceps Tendinopathy. Am. J. Transl. Res..

[24] Martinez M.M., Reif D.R., Pappas D. (2010). Detection of Apoptosis: A Review of Conventional and Novel Techniques. Anal. Methods.

[25] Puzzitiello R.N., Patel B.H., Forlenza E.M., Nwachukwu B.U., Allen A.A., Forsythe B., Salzler M.J. (2020). Adverse Impact of Corticosteroids on Rotator Cuff Tendon Health and Repair: A Systematic Review of Basic Science Studies. Arthrosc. Sports Med. Rehabil..

[26] Poulsen R.C., Watts A.C., Murphy R.J., Snelling S.J., Carr A.J., Hulley P.A. (2014). Glucocorticoids Induce Senescence in Primary Human Tenocytes by Inhibition of Sirtuin 1 and Activation of the P53/P21 Pathway: In Vivo and in Vitro Evidence. Ann. Rheum. Dis..

[27] Dean B.J.F., Franklin S.L., Murphy R.J., Javaid M.K., Carr A.J. (2014). Glucocorticoids Induce Specific Ion-Channel-Mediated Toxicity in Human Rotator Cuff Tendon: A Mechanism Underpinning the Ultimately Deleterious Effect of Steroid Injection in Tendinopathy?. Br. J. Sports Med..

[28] Papazoglu C., Mills A. (2007). P53: At the Crossroad between Cancer and Ageing. J. Pathol..

[29] Itoigawa Y., Yoshida K., Nojiri H., Morikawa D., Kawasaki T., Wada T., Koga A., Maruyama Y., Ishijima M. (2021). Association of Recurrent Tear After Arthroscopic Rotator Cuff Repair and Superoxide-Induced Oxidative Stress. Am. J. Sports Med..

[30] Zhang J.-H., Zhang Y., Herman B. (2003). Caspases, Apoptosis and Aging. Ageing Res. Rev..

[31] Fearon A., Dahlstrom J.E., Twin J., Cook J., Scott A. (2014). The Bonar Score Revisited: Region of Evaluation Significantly Influences the Standardized Assessment of Tendon Degeneration. J. Sci. Med. Sport.

[32] Jaworski Ł., Zabrzyńska M., Klimaszewska-Wiśniewska A., Zielińska W., Grzanka D., Gagat M. (2022). Advances in Microscopic Studies of Tendinopathy: Literature Review and Current Trends, with Special Reference to Neovascularization Process. J. Clin. Med..

[33] Sethi P.M., Sheth C.D., Pauzenberger L., McCarthy M.B.R., Cote M.P., Soneson E., Miller S., Mazzocca A.D. (2018). Macroscopic Rotator Cuff Tendinopathy and Histopathology Do Not Predict Repair Outcomes of Rotator Cuff Tears. Am. J. Sports Med..

[34] Sethi P.M., Miller S.R., Sheth C.D., McCarthy M.B.R., Cote M.P., Mazzocca A.D. (2017). Tendonopathy Does Not Predict Rotator Cuff Healing: Macroscopic Tendon Appearance and Histologic Tendon Scores Do Not Correlate. J. Shoulder Elb. Surg..

[35] Longo U.G., Franceschi F., Ruzzini L., Rabitti C., Morini S., Maffulli N., Denaro V. (2009). Characteristics at Haematoxylin and Eosin Staining of Ruptures of the Long Head of the Biceps Tendon. Br. J. Sports Med..

[36] Riggin C.N., Weiss S.N., Rodriguez A.B., Raja H., Chen M., Schultz S.M., Sehgal C.M., Soslowsky L.J. (2022). Increasing Vascular Response to Injury Improves Tendon Early Healing Outcome in Aged Rats. Ann. Biomed. Eng..

[37] Glait S.A., Mahure S., Loomis C.A., Cammer M., Pham H., Feldman A., Jazrawi L.M., Strauss E.J. (2018). Regional Histologic Differences in the Long Head of the Biceps Tendon Following Subpectoral Biceps Tenodesis in Patients with Rotator Cuff Tears and SLAP Lesions. Knee Surg. Sports Traumatol. Arthrosc..

[38] Lundgreen K., Lian Ø.B., Engebretsen L., Scott A. (2011). Tenocyte Apoptosis in the Torn Rotator Cuff: A Primary or Secondary Pathological Event?. Br. J. Sports Med..

